# Identification of Pyroptosis-Related Gene Signatures Associated with Predicted Immune-Cell Enrichment in Atrial Fibrillation

**DOI:** 10.3390/genes17080858

**Published:** 2026-07-24

**Authors:** Junhao Zhang, Yuwei Ma, Boyang Chen, Li Yu

**Affiliations:** 1 School of Pharmacy, Nanjing University of Chinese Medicine, 138 Xianlin Rd., Nanjing 210023, China; 202411880@njucm.edu.cn (J.Z.); 047122131@njucm.edu.cn (Y.M.); 2 School of Medicine, Putian University, 1133 Xueyuan Road, Chengxiang District, Putian 351100, China

**Keywords:** atrial fibrillation, pyroptosis, predicted immune-cell enrichment, risk prediction, biomarkers

## Abstract

**Background:** Research demonstrates that pyroptosis is a critical factor in the progression of cardiovascular diseases and their related complications. Nevertheless, the precise association between this particular cell death process and the pathophysiological characteristics of atrial fibrillation (AF) remains uncertain. This exploratory study investigates the association between pyroptosis-related genes (PRGs), predicted immune-cell enrichment, and AF using a systems biology approach. **Methods:** Two datasets obtained from the Gene Expression Omnibus, along with a dataset from GeneCards pertaining to pyroptosis-related genes (PRGs), were utilized in this study. Following this, 18 AF-PRGs acquired from the GSE41177 dataset and PRGs were analyzed for functional enrichment using Gene Ontology (GO) annotation, Kyoto Encyclopedia of Genes and Genomes (KEGG) pathway analysis, and gene set enrichment analysis (GSEA). Subsequently, the validation dataset was employed to assess the 18 AF-PRGs, leading to the identification of seven candidate genes for exploratory validation. Then, interaction networks were developed to elucidate potential regulatory relationships among PRGs, miRNAs, transcription factors, and drugs. Finally, single-sample GSEA was used to estimate predicted immune-cell enrichment in AF. **Results:** The KEGG pathway analysis indicated that AF-related PRGs are significantly enriched in pathways linked to NOD-like receptor signaling, lipid metabolism, and bacterial infection, highlighting the potential role of inflammation in the pathogenesis of AF. After validation, we identified seven additional reliable genes: *S100A9*, *S100A4*, *MPEG1*, *NAIP*, *CDK9*, *S100A8*, and *S100A12*. Additionally, 177 miRNAs were predicted to regulate these seven genes, while 50 transcription factors (TFs) were identified to regulate six of them, and 38 drugs were predicted to target six genes as hypothesis-generating interactions. In assessing predicted immune-cell enrichment, we observed significant differences between AF and sinus rhythm atrial tissues. **Conclusion:** This exploratory study suggests that pyroptosis-related inflammatory gene signatures may be associated with AF and provides hypotheses for future mechanistic and translational validation.

## 1. Introduction

Atrial fibrillation (AF) represents the most widespread and clinically significant cardiac arrhythmia on a global scale [1]. As of now, choosing the most effective strategy for managing AF is difficult primarily due to a limited understanding of its underlying causes, which has led to a deficiency in reliable diagnostic tools and treatment options for AF. Additionally, AF can significantly impact a patient’s quality of life due to its association with serious complications, including stroke, heart failure, cognitive decline, and sudden cardiac arrest, which leads to elevated morbidity and mortality rates [2]. Currently, the impact of AF transcends individual health, presenting significant economic challenges to healthcare systems worldwide [3]. The primary approach to treating AF largely relies on catheter ablation. This method has proven to be more effective than anti-arrhythmic medications in preserving sinus rhythm, making it a fundamental component of AF treatment strategies [4]. Current pharmacological therapies for AF primarily target ion channels and demonstrate limited efficacy in preventing the onset or progression of AF in 85% of patients [5]. The lack of effective curative treatments for AF coincides with a limited understanding of the disease’s pathogenesis in each individual patient. Therefore, there is a pressing necessity to identify novel biomarkers and therapeutic targets that can improve clinical outcomes and facilitate more effective management of AF.

Pyroptosis is a form of programmed cell death (PCD) characterized by lysis and inflammation, usually initiated by inflammasomes and carried out by gasdermin proteins [6]. The primary features of pyroptosis include cellular swelling, membrane rupture, and the discharge of intracellular components [7]. Generally, moderate pyroptosis contributes to host defense against pathogen infection; however, excessive pyroptosis can provoke dysregulated inflammatory responses, widespread cell death, and considerable tissue damage, ultimately leading to inflammatory or autoimmune disorders [8]. Further investigation into pyroptosis may enhance the discovery of innovative therapeutic strategies for immunological disorders and a range of other diseases.

Previous studies suggest that pyroptosis-related inflammatory pathways may contribute to AF pathophysiology and its associated complications [9,10]. A study demonstrated that cold exposure in a mouse model increased atrial M1 macrophage enrichment and facilitated cardiac pyroptosis, ultimately resulting in structural remodeling of the atrium [11]. Nonetheless, the precise association between pyroptosis and the pathophysiological mechanisms of AF has yet to be thoroughly elucidated.

Recent advancements in bioinformatics techniques have facilitated extensive analyses of gene expression profiles across diverse disease contexts. By utilizing extensive datasets, researchers are able to identify differentially expressed genes (DEGs) that are associated with specific disease phenotypes. Within the framework of AF, analyses can uncover candidate PRGs that may be prioritized for further biological validation as exploratory biomarkers or mechanistic hypotheses.

## 2. Materials and Methods

In this study, we adopt a systems biology approach to examine the relationship between pyroptosis-related genes (PRGs) and AF. By integrating multi-omics data, including transcriptomic profiles, we seek to identify key PRGs associated with AF and elucidate their biological functions in the context of arrhythmogenesis. The results of this research may facilitate the development of innovative diagnostic and therapeutic strategies targeting the inflammatory pathways implicated in AF.

### 2.1. Microarray Data and Normalization

**Figure 1:**
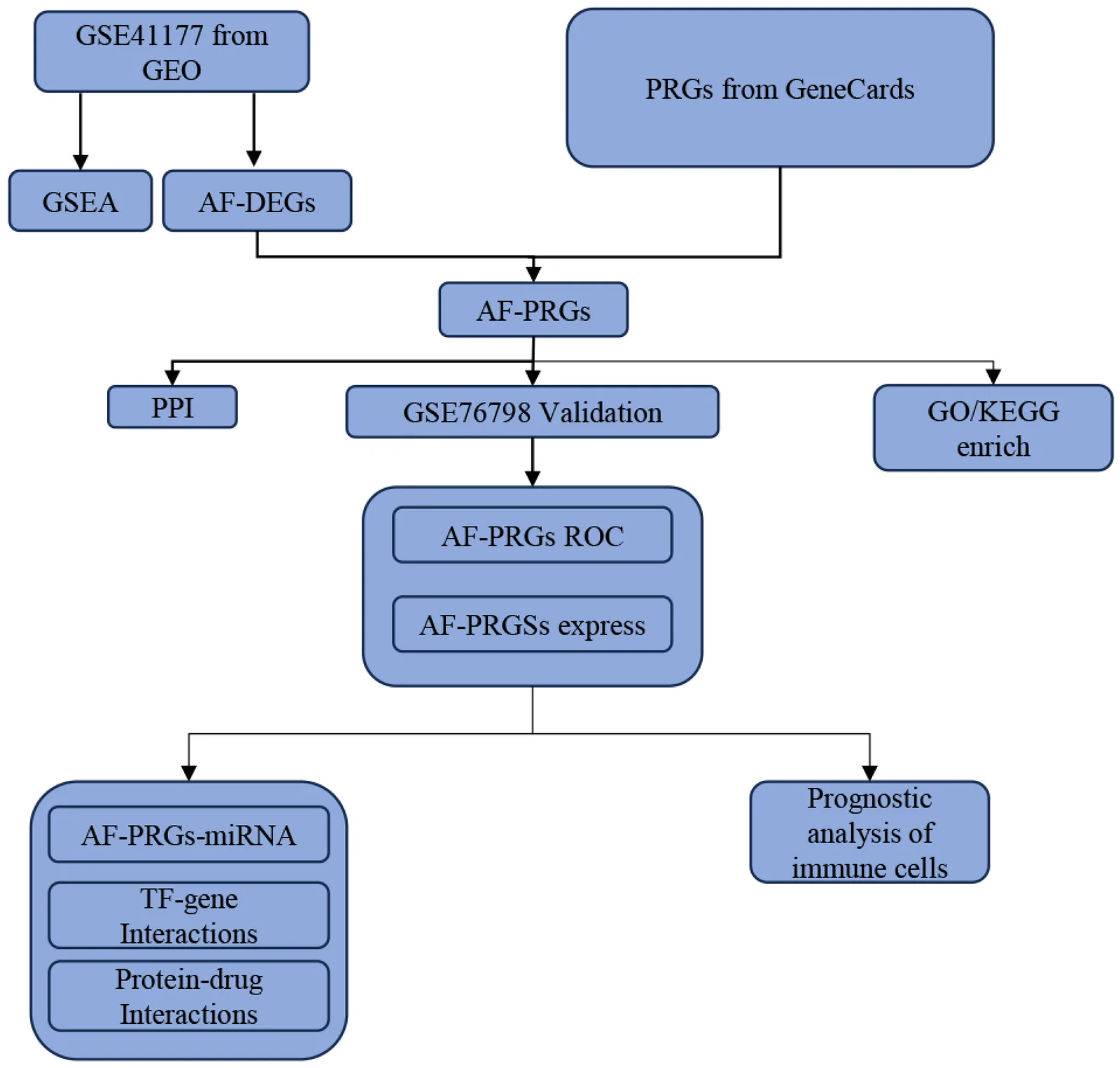
Flow Diagram of the Study.

Figure 1 provides a comprehensive overview of the workflow. GSE41177 [12] and GSE79768 [13] for AF were acquired from the GEO database through the use of the “GEOquery” package (version 2.74.0) in R (version 4.4.2; Bioconductor version 3.20) [14]. In the GSE41177 database, specimens from the paired left atrial-pulmonary vein (LA-PV) junction and the left atrial appendage (LAA) were collected from 16 patients (Homo sapiens) with persistent AF and 3 patients in sinus rhythm who underwent valve surgery. These paired samples were then sent for microarray analysis. In the GSE79768 database, a total of twenty-one paired samples from the left atrium (LA) and right atrium (RA) were collected from patients (Homo sapiens) undergoing cardiac surgery. Among these, thirteen paired samples were subjected to microarray analysis, comprising 6 samples from sinus rhythm and 7 from atrial fibrillation. The GSE79768 served as the validation dataset. The normalization of the data was conducted using the “limma” R package (version 3.62.2) [15]. Principal component analysis (PCA) was then conducted, and the results were visualized using the “ggplot2” package (version 3.5.1).

### 2.2. Identification of DEGs and Acquisition of PRGs

The “limma” R package (version 3.62.2) [15] was utilized to identify differentially expressed genes (DEGs) and assess their differential expression. To control for multiple testing, raw *p*-values were adjusted using the Benjamini–Hochberg false discovery rate (FDR) method. DEGs were defined using the criteria of |log_2_ fold change (FC)| > 1 and FDR-adjusted *p*-value < 0.05. All differential expression analyses and subsequent visualizations were performed based on the FDR-corrected results. The “ggplot2” (version 3.5.1) and “ComplexHeatmap” packages (version 2.22.0) were used to generate volcano plots and heatmaps, respectively [16]. Furthermore, we acquired a total of 593 pyroptosis-related genes (PRGs) from the GeneCards database. The retrieved PRG data were subsequently integrated with DEGs identified between the AF and sinus rhythm groups in the GSE41177 dataset, which were defined as AF-related PRGs (AF-PRGs). The protein-protein interaction (PPI) network for the PRGs was analyzed using the STRING database (http://string-db.org/), followed by visualization with Cytoscape (version 3.10.3) [17].

### 2.3. Functional and Pathway Enrichment Analysis

Functional and pathway enrichment analyses for the AF-PRGs were performed utilizing tools from the Gene Ontology (GO) [18] and Kyoto Encyclopedia of Genes and Genomes (KEGG) [19] databases, respectively. GO analysis is a widely used approach for investigating gene functions, while KEGG offers insights into biological pathways, diseases, and pharmacological information. The data were analyzed using the “clusterProfiler” package (version 4.14.6) in R [20], with a significance threshold established at a *p* < 0.05.

### 2.4. Gene Set Enrichment Analysis

Predefined gene sets from the MSigDB database (https://www.gsea-msigdb.org/gsea/msigdb/collections.jsp, accessed on 19 July 2026) are utilized to assess the distribution patterns of genes within a gene expression profile ranked by their correlation with phenotypes, thereby evaluating their contributions and associations with the phenotype [21]. We obtained the gene sets “c2.cp.kegg.v2022.1.Hs.symbols” and “c5.all.v2022.1.Hs.symbols” from the MSigDB database and conducted GSEA on these sets using the “clusterProfiler” package in R (version 4.14.6) [20], considering a *p* < 0.05 as statistically significant.

### 2.5. Analysis of PRG Expression and ROC Curves

We examined the expression levels of AF-PRGs in affected and control groups using box plots. The Wilcoxon rank-sum test was used for statistical analysis, and the “ggplot2” package was used for data visualization. Receiver operating characteristic (ROC) curves were used to explore the discriminatory performance of these genes within the analyzed datasets. The “pROC” package (version 1.18.5) [22] was used to conduct ROC analysis, and “ggplot2” (version 3.5.1) was used for visualization.

### 2.6. Development of Interaction Networks Among AF-PRGs, Associated microRNAs, Transcription Factors, and Drugs

TarBase v9.0 [23] and ENCODE [24] databases were utilized to identify the miRNAs and transcription factors linked to the differentially expressed AF-PRGs. The miRNA-PRG and TF-PRG interaction networks were visualized using the NetworkAnalyst database platform version 3.0 (version 3.0; https://www.networkanalyst.ca/) [25]. The Drug Gene Interaction Database (DGIdb) version 5.0 (https://www.dgidb.org) is a web-based resource that consolidates data from various drug-gene interaction databases [26]. The drug-PRG interaction network was visualized using Cytoscape (version 3.10.3) [17].

### 2.7. Predicted Immune-Cell Enrichment

Predicted immune-cell enrichment was assessed using the ssGSEA algorithm from the R package GSVA (version 2.0.7) [27], based on 24 immune-cell signatures reported previously [28]. The Wilcoxon rank-sum test was used to compare enrichment scores between the sinus rhythm and AF groups. Spearman correlation analysis was performed to evaluate the association between AF-PRGs and predicted immune-cell enrichment. Correlation heatmaps were generated using ggplot2 to illustrate the relationships between the 24 immune-cell signatures and candidate genes in AF tissues.

### 2.8. Western Blot (WB)

Protein was extracted from left atrial appendage tissue samples of 8 non-atrial fibrillation (non-AF) patients and 8 atrial fibrillation (AF) patients using RIPA lysis buffer (Beyotime Biotechnology, Shanghai, China) containing protease inhibitors. Protein concentration was determined using a BCA assay kit (Thermo Fisher Scientific, Waltham, MA, USA). Equal amounts of protein (30 µg) were separated by 10% SDS-PAGE and transferred to PVDF membranes (MilliporeSigma, Burlington, MA, USA). Membranes were blocked with 5% non-fat milk in TBST for 1 h at room temperature, and then incubated overnight at 4 °C with primary antibodies against S100A4, S100A8, S100A9, S100A12, MPEG1, NAIP, and CDK9. After washing, membranes were incubated with HRP-conjugated secondary antibodies (1:5000; Abcam, Cambridge, UK) for 1 h at room temperature. Protein bands were visualized using enhanced chemiluminescence (ECL) reagents (Thermo Fisher Scientific, Waltham, MA, USA) and quantified with ImageJ software (version 1.54k; National Institutes of Health, Bethesda, MD, USA). Antibodies used for WB are listed in Supplementary Table S2.

### 2.9. Quantitative Real-Time PCR (qPCR)

Total RNA was extracted from left atrial appendage tissue samples of 8 non-AF patients and 32 AF patients using TRIzol reagent (Invitrogen, Carlsbad, CA, USA) following the manufacturer’s instructions. RNA concentration and purity were measured by NanoDrop 2000 (Thermo Fisher Scientific, Waltham, MA, USA). cDNA was synthesized using a PrimeScript RT kit (Takara Bio Inc., Kusatsu, Japan). qPCR was performed using SYBR Green Master Mix (Applied Biosystems, Foster City, CA, USA) on a QuantStudio 6 Flex system (Applied Biosystems, Foster City, CA, USA). The relative expression of *S100A4*, *S100A8*, *S100A9*, *S100A12*, *MPEG1*, *NAIP*, and *CDK9* was calculated using the 2^−ΔΔCt^ method with *GAPDH* as the internal control. Primer sequences are listed in Supplementary Table S1.

### 2.10. Patient Characteristics

The clinical characteristics of all patient groups are summarized in Table 1 and Table 2. Left atrial appendage (LAA) specimens from the control group were obtained from patients with valvular heart disease who were in sinus rhythm and underwent prophylactic LAA resection during cardiac surgery; these patients had no severe underlying systemic diseases or inflammation-related disorders. LAA tissues from the persistent atrial fibrillation (AF) group were collected from patients with persistent AF without other major underlying diseases or inflammatory conditions. For the AF follow-up cohort, recurrence was defined according to standard electrocardiographic or Holter monitoring criteria. No significant differences were observed between groups in baseline characteristics, including age, sex, and common cardiovascular comorbidities. Detailed clinical information for all groups is provided in the Supplementary Tables.

## 3. Results

### 3.1. Bioinformatics Analysis

#### 3.1.1. Analysis of Differential Gene Expression

**Figure 2:**
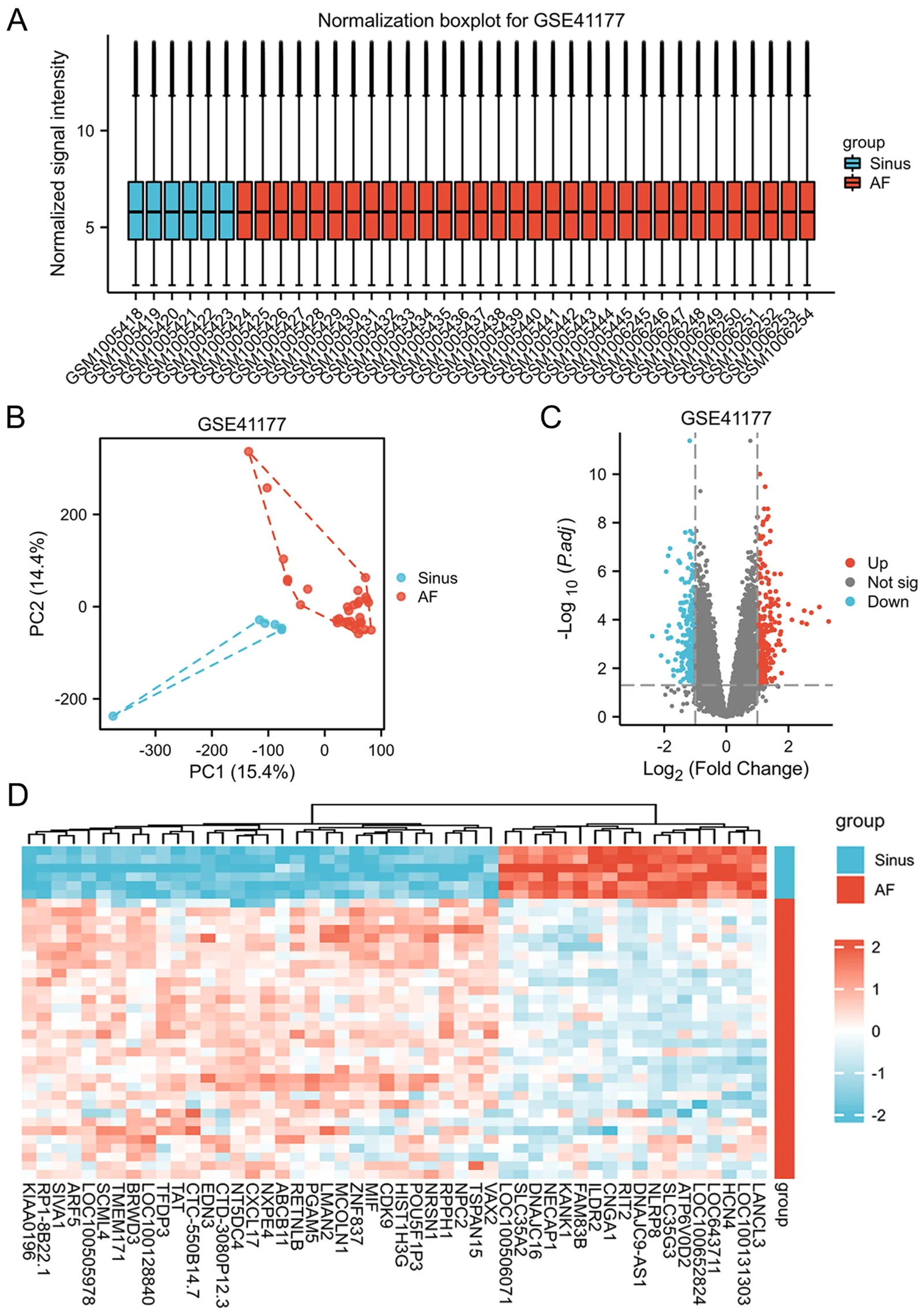
Analysis of Differential Gene Expression. (**A**) Normalized box plots of the dataset samples for GSE41177. (**B**) Principal component analysis (PCA) plots for GSE41177. Blue and red boxes or dots represent the sinus rhythm group and the AF group, respectively. (**C**) Volcano plot depicting DEGs between sinus rhythm and atrial fibrillation (AF) in GSE41177. Blue and red indicate downregulated and upregulated genes, respectively, while grey nodes indicate genes that were not significantly differentially expressed. The vertical dashed lines indicate log_2_ (fold change) = −1 and 1, and the horizontal dashed line indicates an FDR-adjusted *p*-value of 0.05. DEGs were defined as genes with |log_2_ fold change (FC)| > 1 and FDR-adjusted *p*-value < 0.05. (**D**) Heatmaps of AF-related DEGs in GSE41177. The vertical axis represents the respective differentially expressed genes (DEGs), while the horizontal axis denotes patient IDs. Blue indicates low gene expression, red signifies high gene expression, blue bars represent sinus rhythm tissue, and red bars denote atrial fibrillation (AF) tissue.

**Figure 3:**
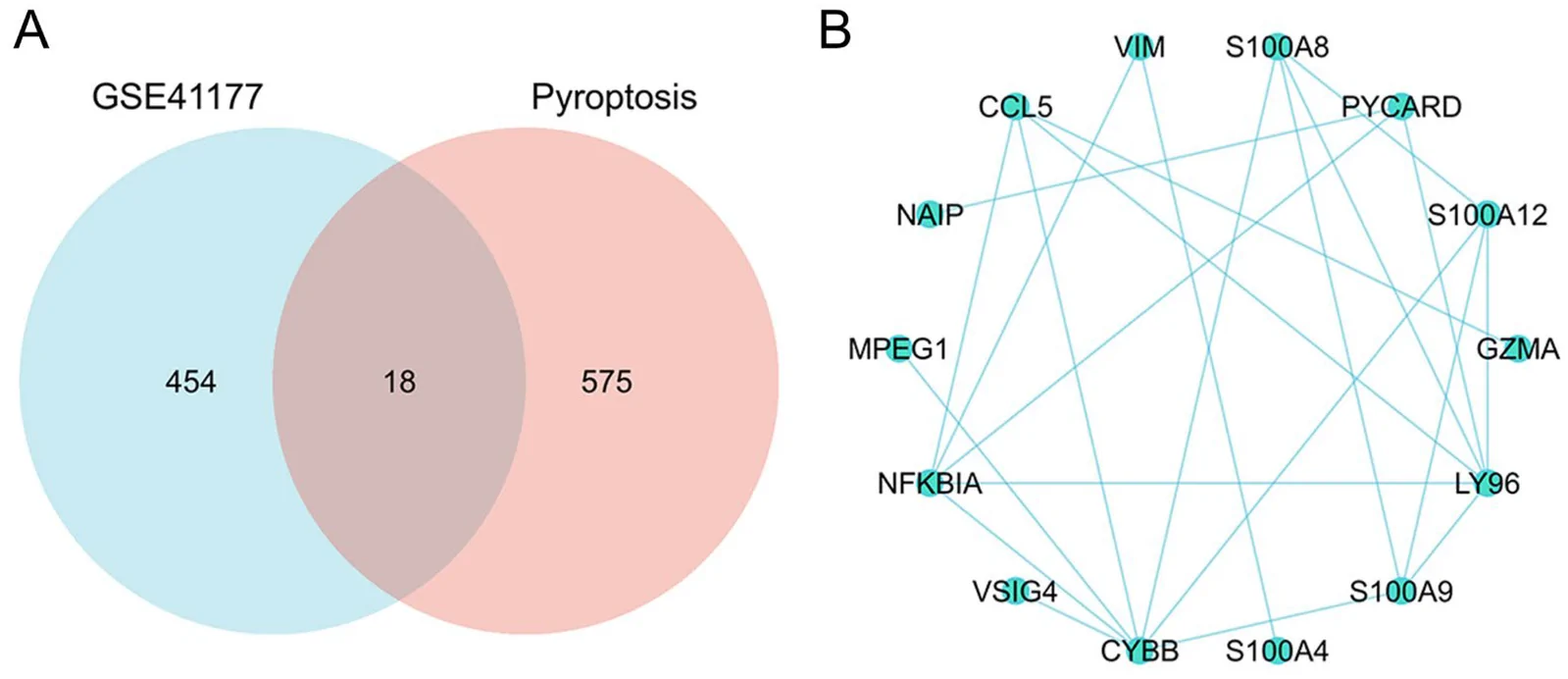
AF-Related PRGs. (**A**) The intersection of differentially expressed genes (DEGs) and pyroptosis-related genes. A total of eighteen differentially expressed genes (DEGs) associated with atrial fibrillation (AF) and pyroptosis were identified: *S100A8*, *S100A12*, *S100A9*, *S100A4*, *CYBB*, *LY96*, *MPEG1*, *NAIP*, *CPA3*, *NFKBIA*, *PYCARD*, *CCL5*, *CDK9*, *VIM*, *TUBB6*, *VSIG4*, *MCOLN1*, and *GZMA*. (**B**) Diagram of the protein-protein interaction (PPI) network for AF-PRGs, created using Cytoscape software and based on data from the STRING database.

At the outset, normalization of the sample data from the GSE41177 dataset was conducted, subsequently leading to the generation of box plots for the normalized data (Figure 2A). Principal component analysis (PCA) was used to display the differences of sinus rhythm group and atrial fibrillation (AF) group (Figure 2B). The horizontal axis represents the first principal component of PCA dimensionality reduction, and the vertical axis represents the second principal component of PCA dimensionality reduction. The numbers in parentheses represent the proportion of the principal component explained. In order to assess how gene expression levels affect AF tissues compared to sinus rhythm tissues, we utilized the differential analysis package “limma [15]” to identify differentially expressed genes (DEGs) in the GSE41177 dataset, which was subsequently illustrated using volcano plots (Figure 2C). In the GSE41177 dataset, we identified 472 differentially expressed genes (DEGs), of which 262 were upregulated and 210 were downregulated. A heatmap for classification was created to clearly illustrate these findings (Figure 2D). Furthermore, by combining the PRGs with the DEGs from the GSE41177 dataset, we identified 18 genes associated with AF that are common to both DEGs and PRGs (Figure 3A). The following is a list of 18 genes: *S100A9*, *S100A4*, *CYBB*, *LY96*, *MPEG1*, *NAIP*, *CPA3*, *NFKBIA*, *PYCARD*, *CCL5*, *CDK9*, *VIM*, *TUBB6*, *VSIG4*, *MCOLN1*, *GZMA*, *S100A8*, *S100A12*.

#### 3.1.2. Protein-Protein Interaction Network

In this research, the AF-PRGs sourced from the STRING database were employed to build a protein-protein interaction (PPI) network of the differentially expressed AF-PRGs (Figure 3B). The diagram demonstrates that the three genes with the most significant cooperative interactions are *CYBB*, *LY96*, and *NFKBIA*.

#### 3.1.3. Functional Enrichment Analysis of Differentially Expressed AF-Related PRGs

**Figure 4:**
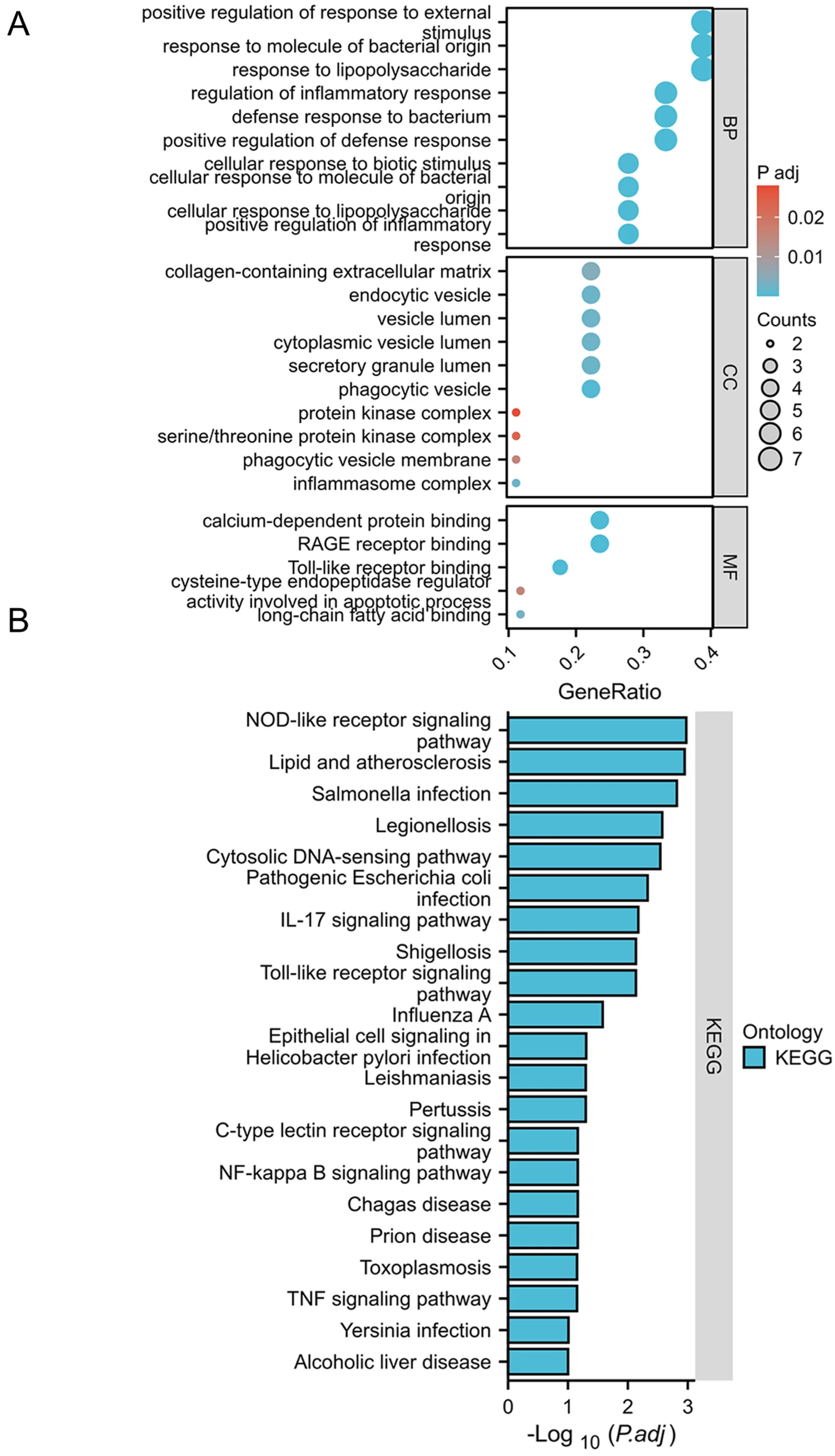
Functional Enrichment Analysis of Differentially Expressed AF-Related PRGs. (**A**) Gene Ontology (GO) enrichment analysis. Gene Ontology (GO) enrichment analysis classifies gene functions into three main groups: biological processes (BP), cellular components (CC), and molecular functions (MF). (**B**) KEGG pathway enrichment analysis.

Analysis of functional enrichment for the differentially expressed genes revealed several biological processes (GO: biological process, GO: cellular component, and GO: molecular function) (Figure 4A). The KEGG annotation of the AF-PRGs indicated a significant enrichment in pathways associated with NOD-like receptor signaling pathway, lipid and atherosclerosis, Salmonella infection, cytosolic DNA-sensing pathway, and so forth (Figure 4B). The differentially expressed AF-PRGs were primarily engaged in response to lipopolysaccharide, response to molecules of bacterial origin, positive regulation of inflammatory response, positive regulation of response to external stimulus, positive regulation of defense response, and other biological processes (Figure 4A). They exhibited enrichment in cellular components including phagocytic vesicle, inflammasome complex, secretory granule lumen, cytoplasmic vesicle lumen, and so forth (Figure 4A). In terms of molecular functions, they exhibited a high abundance for the GO terms associated with RAGE receptor binding, Toll-like receptor binding, calcium-dependent protein binding, long-chain fatty acid binding, and cysteine-type endopeptidase regulator activity involved in the apoptotic process (Figure 4A).

#### 3.1.4. Gene Set Enrichment Analysis

**Figure 5:**
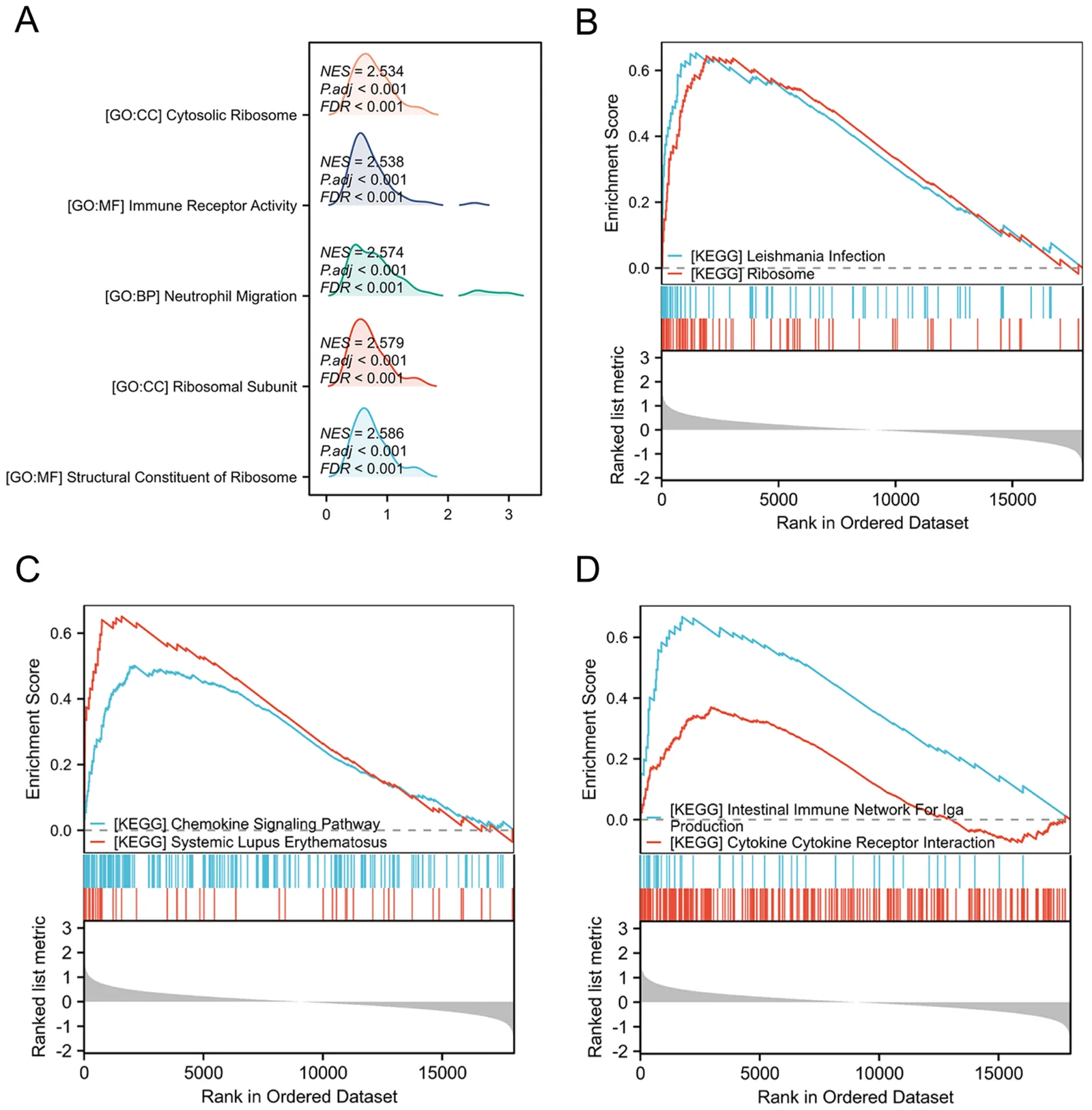
GSEA-GO and GSEA-KEGG Analyses of GSE41177 Data. (**A**) GSEA–GO analysis of GSE41177 data. (**B**–**D**) GSEA–KEGG analysis of GSE41177 data.

To assess the influence of gene expression levels on AF, Gene Set Enrichment Analysis (GSEA) was conducted on the GSE41177 dataset to elucidate the relationships between gene expression and associated biological processes, cellular components, and molecular functions. The analysis demonstrated that within the GSE41177 dataset, the genes most profoundly affected the structural constituent of the ribosome, ribosomal subunit, neutrophil migration, immune receptor activity, and cytosolic ribosome (Figure 5A). GSEA-KEGG analysis of the GSE41177 dataset revealed that the enriched pathways were associated with Leishmania infection, ribosome, chemokine signaling pathway, systemic lupus erythematosus, intestinal immune network for IgA production, cytokine-cytokine receptor interaction, and antigen processing and presentation (Figure 5B–D).

#### 3.1.5. AF-PRG Expression Analysis and ROC Validation

**Figure 6:**
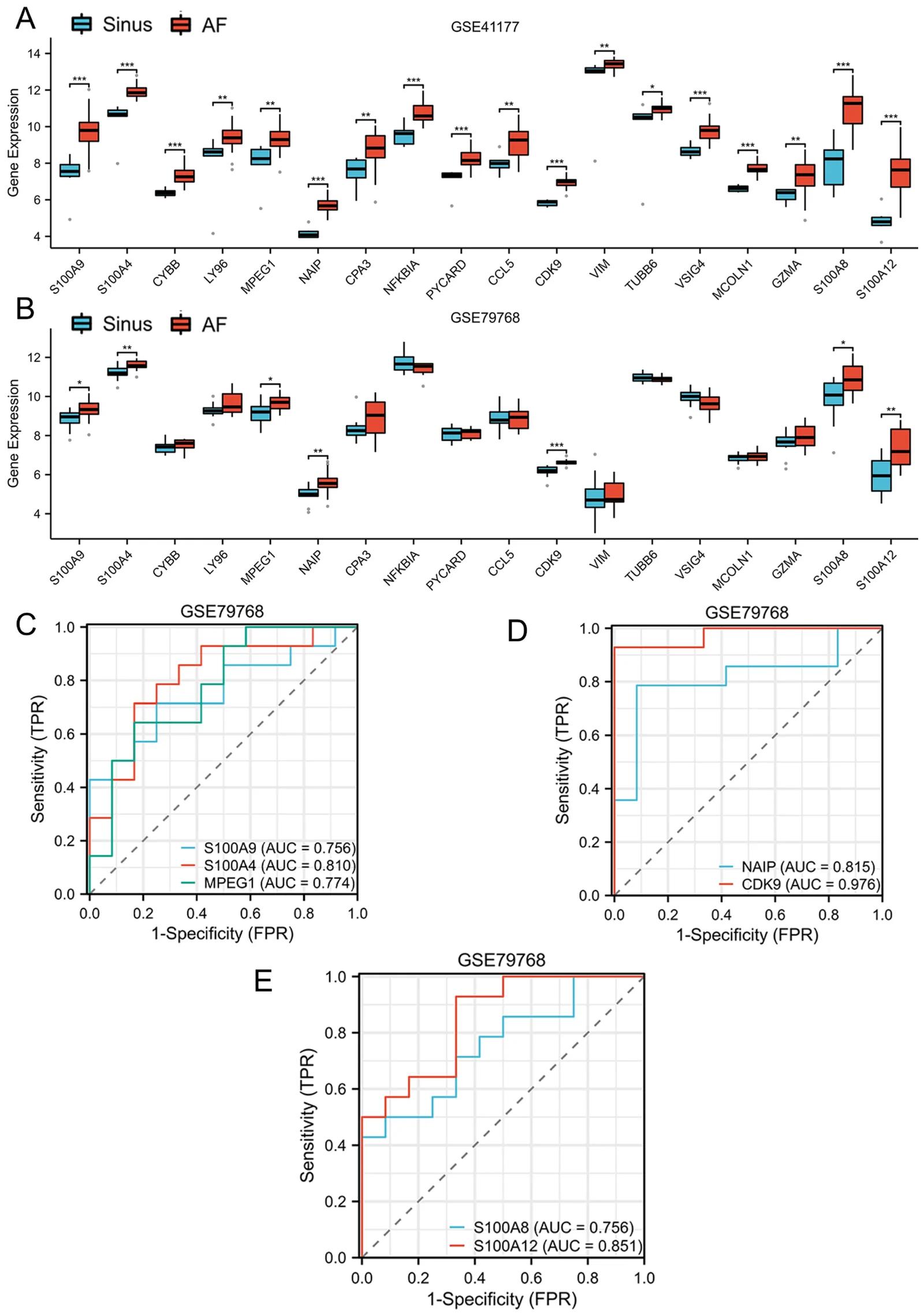
Expression Levels of Atrial Fibrillation-Associated Pyroptosis-Related Genes (AF-PRGs). (**A**) Expression levels of the 18 atrial fibrillation-associated pyroptosis-related genes (AF-PRGs) in the GSE41177 dataset are presented, with blue boxes indicating the sinus rhythm group and red boxes representing the AF group. Statistical significance is indicated as follows: *, *p* < 0.05; **, *p* < 0.01; ***, *p* < 0.001. (**B**) Expression levels of the 18 atrial fibrillation-associated pyroptosis-related genes (AF-PRGs) in the GSE79768 dataset are presented, with blue boxes indicating the sinus rhythm group and red boxes representing the AF group. (*, *p* < 0.05; **, *p* < 0.01; ***, *p* < 0.001). (**C**–**E**) Receiver operating characteristic (ROC) curve analysis for 7 atrial fibrillation-related genes (AF-PRGs) in the GSE79768 dataset (AUC > 0.7).

To reduce potential overfitting bias and further evaluate the diagnostic robustness of the selected genes, ROC curve analysis was performed in the independent external validation dataset GSE79768. The results showed that all seven genes retained diagnostic value for distinguishing AF from control samples, with AUC values ranging from 0.756 to 0.976. Among them, *CDK9* showed the best diagnostic performance, with an AUC of 0.976 and a 95% CI of 0.925–1.000, followed by S100A12 with an AUC of 0.851 and a 95% CI of 0.705–0.998, *NAIP* with an AUC of 0.815 and a 95% CI of 0.636–0.995, and S100A4 with an AUC of 0.810 and a 95% CI of 0.635–0.985. The optimal cut-off values, sensitivity, specificity, and accuracy were also calculated. Notably, *CDK9* achieved the highest overall accuracy, with a cut-off value of 6.4972, sensitivity of 0.92857, specificity of 1.00000, and accuracy of 0.96154. These results suggest that the selected genes, particularly *CDK9*, *S100A12*, *NAIP*, and *S100A4*, maintain stable diagnostic performance in an independent validation cohort (Figure 6).

#### 3.1.6. Network Analysis of AF-PRGs, Associated miRNAs, Transcription Factors, and Drugs

**Figure 7:**
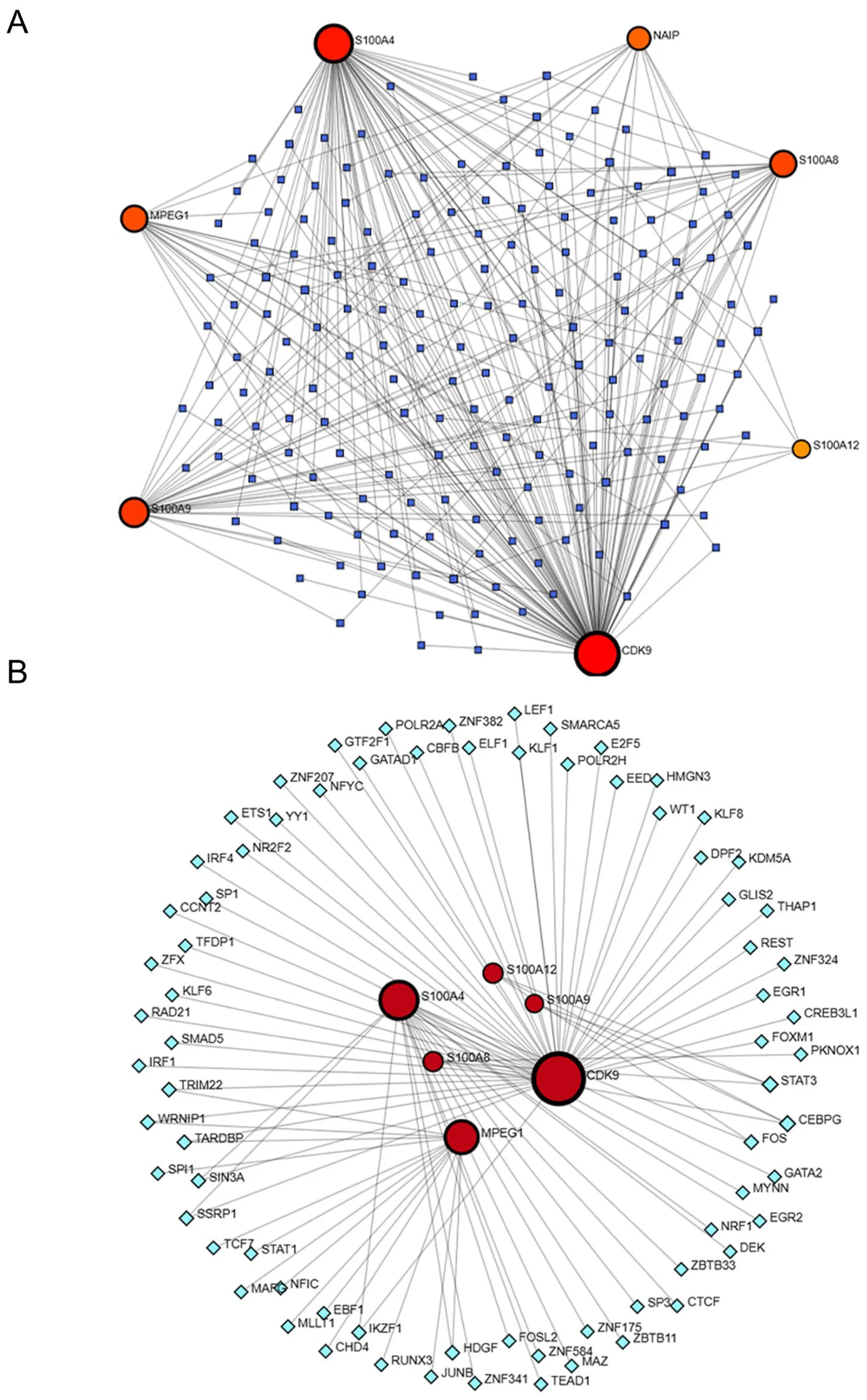

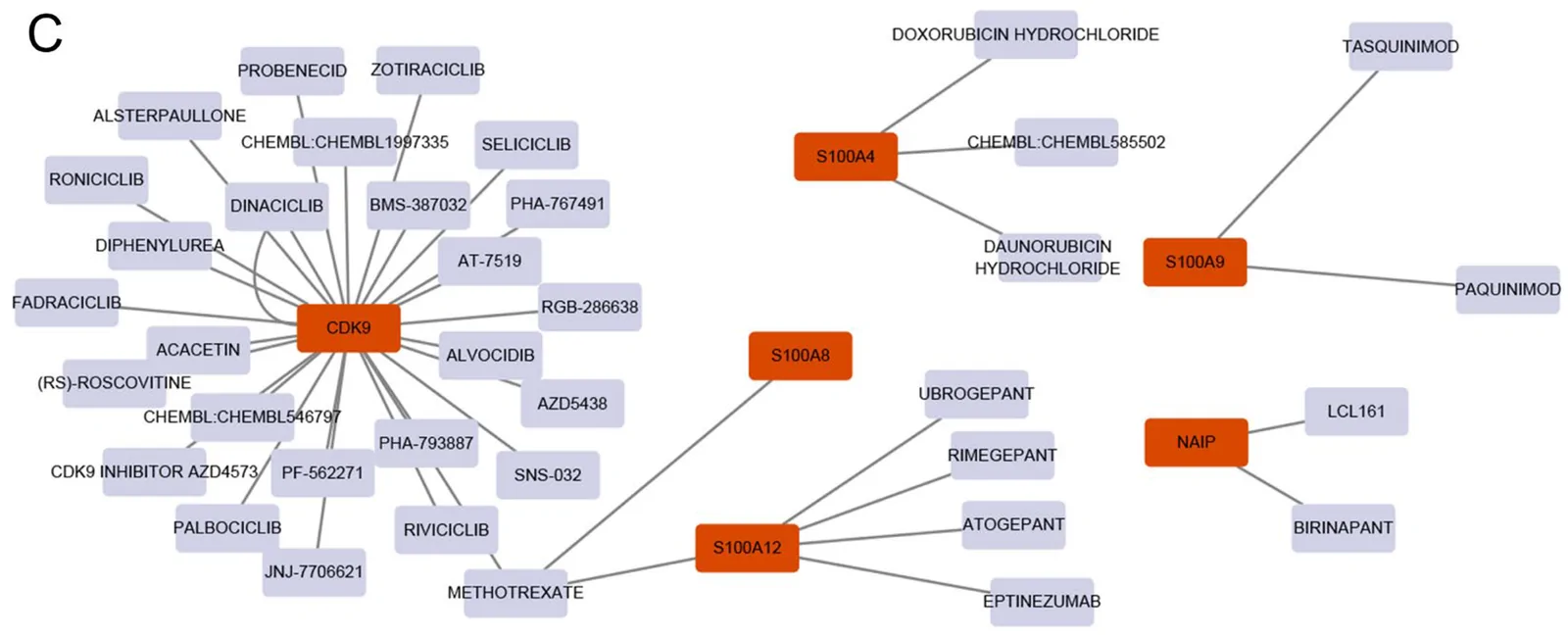
Network Visualization Depicting the Interactions of Atrial Fibrillation-Associated Pyroptosis-Related Genes (AF-PRGs) with miRNAs, Transcription Factors (TFs), and Drugs. (**A**) Network diagram illustrating the interactions between AF-PRGs and miRNAs, with blue nodes representing miRNAs and other nodes denoting AF-PRGs. (**B**) Network diagram depicting the interactions between AF-PRGs and transcription factors (TFs), with green nodes representing TFs and other nodes denoting AF-PRGs. (**C**) Relationship between atrial fibrillation-associated pyroptosis-related genes (AF-PRGs) and drugs. The purple nodes denote drugs, while the orange nodes represent AF-PRGs.

We developed an interaction network between AF-PRGs and miRNAs that includes seven genes and 177 miRNAs (Figure 7A). The five foremost predictions of the AF-PRG-miRNA interaction network included *CDK9*, which was targeted by 110 miRNAs; *S100A4*, which was targeted by 64 miRNAs; *S100A9*, which was targeted by 28 miRNAs; *S100A8*, which was targeted by 20 miRNAs; *MPEG1*, which was targeted by 20 miRNAs. Additionally, the analysis revealed that miRNAs hsa-miR-20a-5p and hsa-miR-671-5p are concurrently associated with the genes *S100A4*, *S100A8*, *S100A9*, and *S100A12*. The AF-PRG-TF interaction network included six genes and 50 transcription factors (Figure 7B). The five foremost predictions of the AF-PRG-TF interaction network included *CDK9* (targeted by 44 TFs), *S100A4* (targeted by 20 TFs), *MPEG1* (targeted by 14 TFs), *S100A12* (targeted by 3 TFs), and *S100A8* (targeted by 3 TFs). Additionally, the chart demonstrates that transcription factors CEBPG and STAT3 are concurrently associated with the three genes *S100A8*, *S100A9*, and *S100A12*. The AF-PRG-drug interaction network comprised 38 drugs and six genes, as depicted in Figure 7C. Methotrexate was predicted by DGIdb to interact with CDK9, S100A8, and S100A12.

#### 3.1.7. Association Between Predicted Immune-Cell Enrichment and AF

**Figure 8:**
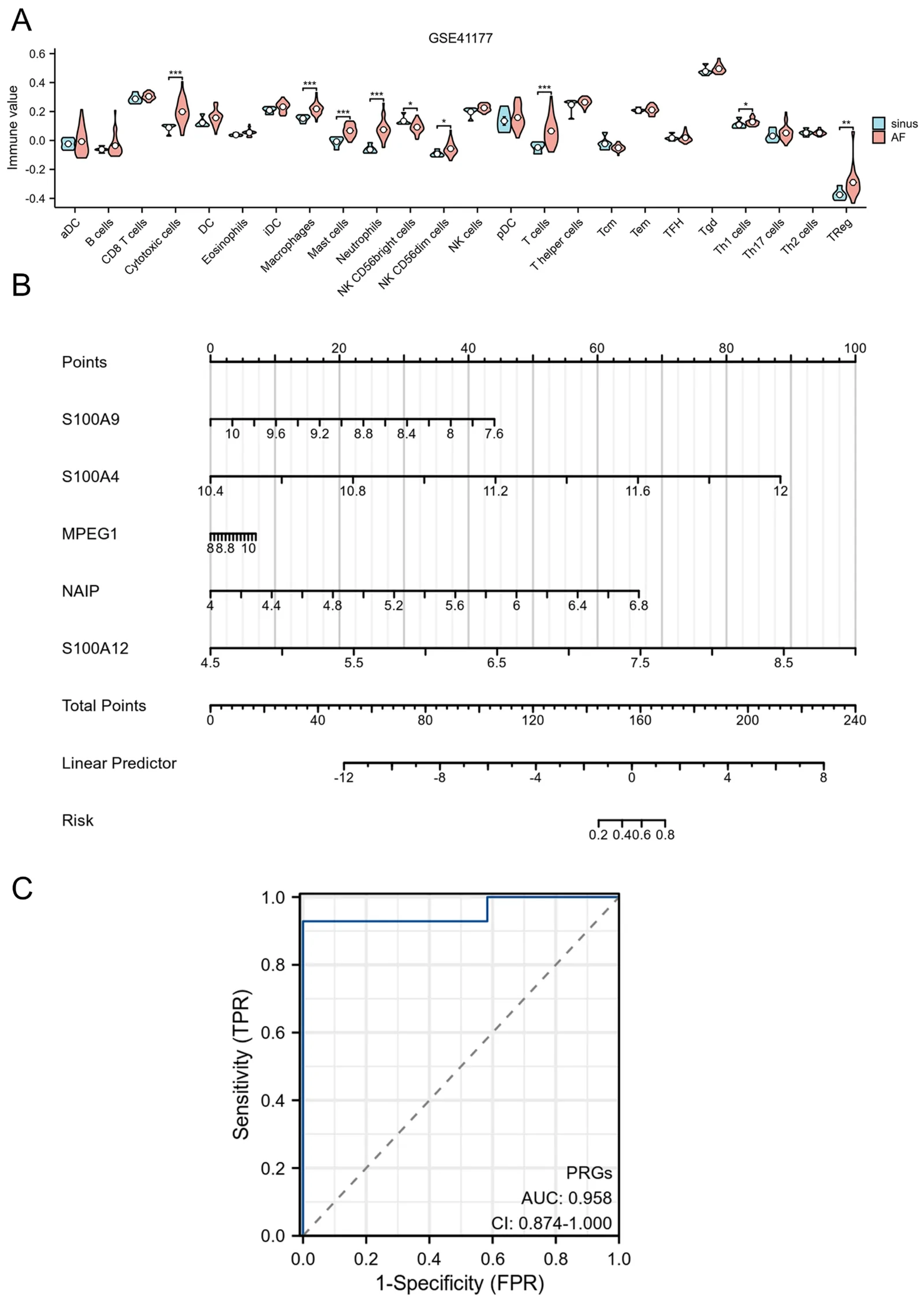
Predicted Immune-Cell Enrichment and Nomogram Analysis. (**A**) Variations in the enrichment levels of 24 immune-cell signatures within the GSE41177 dataset. (*, *p* < 0.05; **, *p* < 0.01; ***, *p* < 0.001). (**B**) Nomogram based on S100A9, S100A4, MPEG1, NAIP, and S100A12 for predicting AF risk in the GSE79768 dataset. (**C**) ROC curve evaluating the diagnostic performance of the pyroptosis-related gene signature (PRGs) in the GSE79768 dataset.

To further investigate immune-related alterations in AF, we applied ssGSEA to estimate the predicted enrichment of 24 immune cell subpopulations in the GSE41177 dataset. The violin plot showed significant differences in predicted immune-cell enrichment between AF and sinus rhythm samples. Compared with sinus rhythm tissues, AF samples showed higher predicted enrichment of cytotoxic cells, macrophages, mast cells, neutrophils, T cells, Th1 cells, and Treg cells, suggesting an inflammatory immune-related transcriptional profile in AF (Figure 8A).

Based on the selected pyroptosis-related genes, including *S100A9*, *S100A4*, *MPEG1*, NAIP, and S100A12, we constructed a nomogram model to predict AF risk. In this model, each gene was assigned a specific score according to its expression level, and the total score was used to estimate the individual probability of AF (Figure 8B). Receiver operating characteristic curve analysis was subsequently performed to assess the diagnostic performance of the model. The gene-based model showed excellent discriminative ability, with an area under the curve of 0.958 and a 95% confidence interval of 0.874–1.000 (Figure 8C). These findings suggest that pyroptosis-related genes are closely associated with predicted immune-cell enrichment in AF and may serve as potential biomarkers for AF risk prediction.

**Figure 9:**
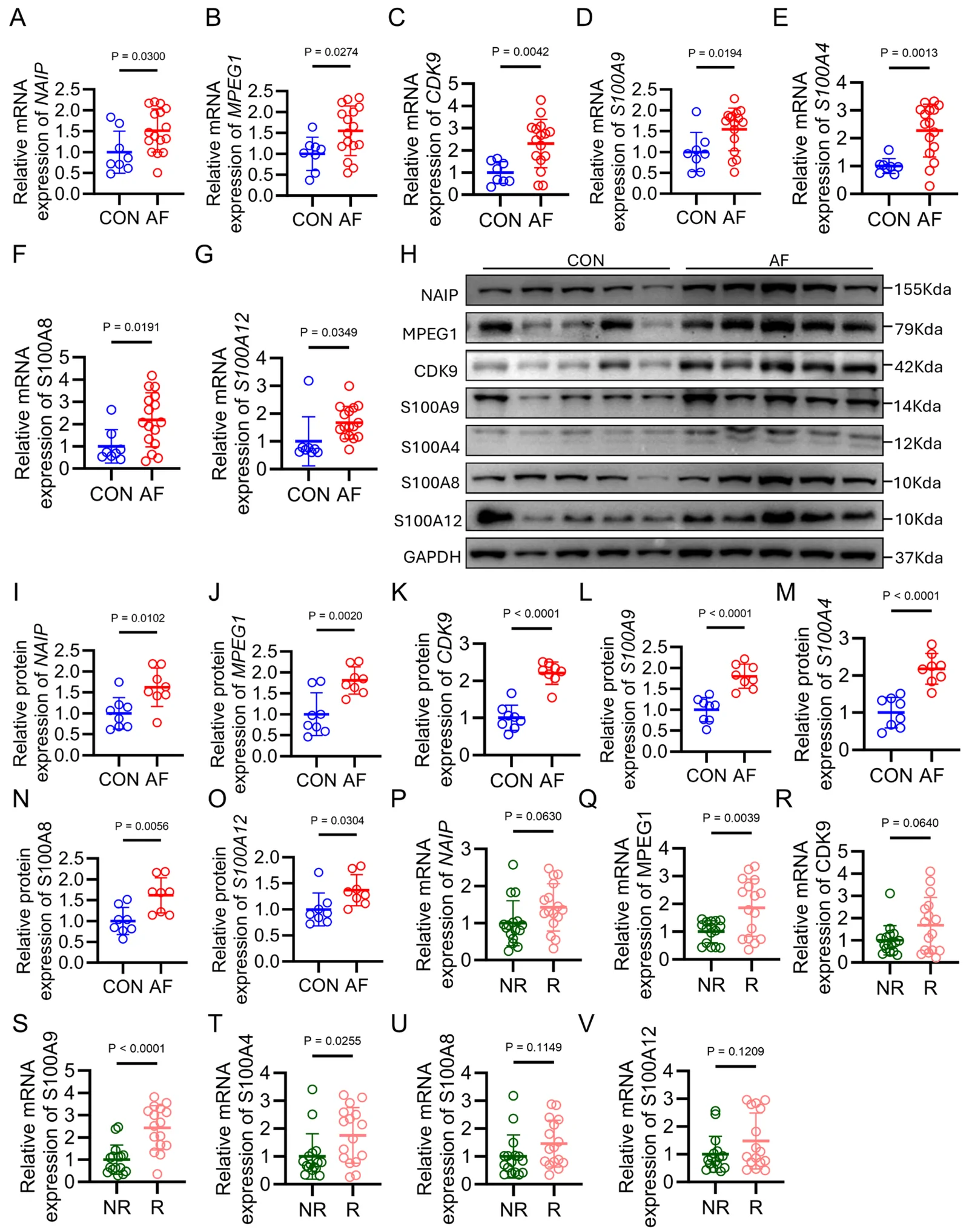
Clinical sample validation of the seven related genes. (**A**–**O**) Representative Western blot images, quantitative protein analysis, and RT–qPCR validation of *S100A9*, *S100A4*, *MPEG1*, *NAIP*, *CDK9*, *S100A8*, and *S100A12* expression in left atrial appendage (LAA) tissues from control and atrial fibrillation (AF) patients. (**P**–**V**) RT–qPCR analysis of *S100A9*, *S100A4*, *MPEG1*, *NAIP*, *CDK9*, *S100A8*, and *S100A12* mRNA expression in LAA tissues from patients with recurrent and non-recurrent AF after surgery.

To further validate the expression patterns of the pivotal genes in AF, we examined their mRNA and protein levels in clinical samples. Quantitative real-time PCR showed that the mRNA expression levels of *NAIP*, *MPEG1*, *CDK9*, *S100A9*, *S100A4*, *S100A8*, and *S100A12* were significantly higher in AF tissues than in control tissues (Figure 9A–G). Consistently, Western blot analysis demonstrated increased protein expression of these genes in AF samples (Figure 9H). Quantitative analysis further confirmed that the protein levels of NAIP, MPEG1, CDK9, S100A9, S100A4, S100A8, and S100A12 were significantly elevated in the AF group compared with the control group (Figure 9I–O). These results indicate that the identified pivotal genes were upregulated at both the transcriptional and translational levels in AF. We further performed an exploratory analysis of postoperative AF recurrence. Compared with patients without recurrence, patients with recurrent AF showed higher mRNA expression levels of MPEG1, S100A9, and S100A4 (Figure 9Q,S,T). However, given the limited sample size, incomplete follow-up information, and lack of Cox regression or recurrence-specific ROC analysis, these findings should be interpreted as preliminary associations rather than validated recurrence predictors.

## 4. Discussion

With the global increase in life expectancy, the significant rise in AF prevalence among the general population is emerging as a pressing public health concern [2]. Additionally, the emergence of new atrial fibrillation during critical illness is associated with increased risks of stroke, heart failure, and mortality [29]. Pyroptosis is a form of programmed cell death (PCD) linked to inflammatory responses [30]. PCD is characterized as the autonomous and orderly elimination of cells regulated by genetic mechanisms to maintain homeostasis, a process that can be obstructed by inhibitors of cellular signaling pathways [31,32]. Previous research has demonstrated that pyroptosis is crucial in the development of various cardiovascular diseases, involving cell types such as endothelial cells and vascular smooth muscle cells, among others [33,34]. Pyroptosis is a meticulously controlled form of cell death, and its inhibition through pharmacological or genetic means offers cardioprotective effects in various situations [33]. Nonetheless, there is insufficient research regarding the potential involvement of pyroptosis in the etiology and progression of AF and its related complications. In this study, we investigated AF-associated pyroptosis-related gene (PRG) signatures, with a particular focus on their links to inflammatory processes that may contribute to disease pathophysiology. By integrating transcriptomic data through a systems biology approach, we aimed to identify candidate PRGs potentially involved in AF. These findings provide a foundation for generating hypotheses and guiding future mechanistic and translational studies targeting inflammatory pathways in AF, while acknowledging that further experimental validation is required to establish causality and clinical relevance. A major biological limitation of this study is the source of atrial tissue samples. Both GEO datasets and clinical samples were derived from surgical populations with structural cardiovascular disease. Therefore, the observed transcriptional differences may not be specific to AF itself, but may also reflect valvular disease, atrial remodeling, heart failure-related changes, aging, medication exposure, inflammatory comorbidities, or surgical selection bias. Thus, these findings should be interpreted as AF-associated rather than AF-specific transcriptional changes.

In our analysis, we identified 472 differentially expressed genes (DEGs) from the GSE41177 dataset, effectively distinguishing patients with atrial fibrillation (AF) from those in sinus rhythm. Among these, 18 pyroptosis-related genes (PRGs), including *S100A9*, *S100A4*, and *CYBB*, were significantly upregulated in AF samples compared with controls. The differential expression of these genes suggests potential involvement in inflammatory and pyroptosis-associated pathways relevant to AF pathophysiology, highlighting avenues for further investigation. Previous studies have established that inflammation plays a key role in the initiation and maintenance of AF [35], reinforcing the rationale for exploring these PRGs as candidate biomarkers and potential targets in future mechanistic and translational research.

The pathway analysis demonstrated that the identified atrial fibrillation-related PRGs are significantly enriched in several critical biological pathways, including the NOD-like receptor signaling pathway, lipid metabolism, and atherosclerosis. This indicates a complex interaction between immune responses and metabolic changes within the context of AF. The NOD-like receptor signaling pathway is recognized for its role in mediating inflammatory responses and has been associated with various cardiac conditions, thereby reinforcing the significance of inflammation in the development of AF [36,37]. Furthermore, the correlation of these genes with lipid metabolism and atherosclerosis highlights the critical role of metabolic dysregulation within the cardiovascular system, which may play a significant part in the onset of AF [38]. Exploring these pathways in relation to patient outcomes may yield valuable insights into the prognostic significance of these PRGs and their potential as therapeutic intervention targets. It should be emphasized that this study inferred pyroptosis-related signatures from transcriptomic data and public PRG annotations, but did not directly demonstrate pyroptotic cell death. Classical pyroptosis markers, including NLRP3 activation, caspase-1 activity, cleaved gasdermin D, ASC speck formation, and IL-1β/IL-18 release, were not measured. Therefore, our findings should be interpreted as pyroptosis-related inflammatory transcriptional signatures rather than direct evidence of active pyroptosis in AF tissues. Although NLRP3 inflammasome activation has been strongly implicated in AF, NLRP3 itself was not retained as a candidate gene in our screening. This may be due to post-transcriptional regulation of inflammasome activity, bulk tissue heterogeneity, limited sample size, microarray platform limitations, and strict DEG selection criteria.

In our differential analysis, we consistently identified that *S100A9*, *S100A4*, *MPEG1*, *NAIP*, *CDK9*, *S100A8*, and *S100A12* were significantly upregulated in both the primary and validation datasets.

The reproducible expression patterns of these genes across independent cohorts support their prioritization as candidate AF-associated markers for further study. Their discriminatory capacity in tissue-based analyses also indicates possible involvement in AF-related pathological processes, although validation in larger and clinically diverse populations will be necessary to determine the robustness and broader applicability of these observations. Moreover, network analysis of AF-associated PRGs revealed predicted interactions with multiple miRNAs, transcription factors, and pharmacological compounds, reflecting the potentially intricate regulatory mechanisms underlying AF. While these interaction networks may provide a useful framework for future hypothesis-driven research, their biological significance and potential clinical implications require confirmation through additional experimental and translational studies. In line with these results, RT–qPCR analysis demonstrated increased mRNA expression of *S100A9*, *S100A4*, and *MPEG1* in left atrial appendage tissues from patients with recurrent AF compared with non-recurrent cases. Nevertheless, the relatively limited sample size and absence of mechanistic validation warrant cautious interpretation of these associations. *CDK9* should be interpreted cautiously because it is not a classical pyroptosis executor. As a transcriptional kinase involved in RNA polymerase II-dependent transcriptional elongation, *CDK9* may reflect transcriptional remodeling associated with AF or structural heart disease rather than direct pyroptotic cell death.

S100 proteins are calcium-binding molecules that are uniquely found in vertebrate species. These proteins are defined by the presence of two EF hands, which house the calcium-binding domains [39]. Furthermore, certain members of the S100 family have demonstrated the ability to bind Zn^2+^ and Cu^2+^, indicating that their biological activity in some instances may be modulated by Zn^2+^ and/or Cu^2+^, instead of Ca^2+^ [40]. Prior research in cardiovascular disease has indicated that *S100A1* influences not only systolic (contracting) cytoplasmic calcium levels but also the regulation of diastolic (resting) calcium [41]. In clinical investigations, multiple studies have identified serum levels of the S100 protein family as prognostic indicators following cardiac arrest [42,43]. Concurrently, the S100 protein family has been demonstrated to play a pivotal role in the onset and progression of various diseases through inflammatory processes [44,45,46]. In our investigation, four members of the S100 protein family were ultimately prioritized as pyroptosis-related and AF-associated candidates. Consequently, our future research will comprehensively investigate the role of S100 protein family members S100A9, S100A4, S100A8, and S100A12 in AF pathobiology and assess whether their mechanisms are linked to inflammasome activation and pyroptosis. Although this study focused on inflammation-related signatures, AF is maintained by multiple mechanisms, including atrial fibrosis, conduction slowing, connexin remodeling, calcium-handling abnormalities, oxidative stress, and autonomic dysfunction. The identified inflammatory genes may interact with these established remodeling processes, but they should not be interpreted as the sole mechanism underlying AF.

The prominent upregulation of S100A8, S100A9, and S100A12 is particularly noteworthy because these proteins are recognized mediators of innate immune activation, neutrophil activation, oxidative stress, and inflammatory amplification. In the cardiovascular system, S100A8/A9 signaling has been linked to myocardial fibrosis, endothelial dysfunction, fibroblast activation, and adverse cardiac remodeling [44]. These processes are mechanistically relevant to AF because atrial fibrosis and structural remodeling provide a substrate for conduction heterogeneity, while oxidative stress, inflammatory cytokine signaling, calcium-handling abnormalities, and autonomic remodeling can further facilitate electrical remodeling and AF maintenance [46]. Therefore, our findings support the hypothesis that S100-family-associated inflammatory signatures may participate in atrial arrhythmogenic remodeling; however, this hypothesis requires direct experimental validation.

Investigating immune-cell signatures and their interactions with these gene products in AF patients may provide deeper insights into the pathophysiology of the arrhythmia and identify novel therapeutic targets. However, this study also has limitations that warrant careful consideration. First, the sample size employed in the analysis may limit the generalizability of our findings. Second, the included tissue samples were derived from surgical populations, and the available public datasets lacked detailed clinical phenotyping, including AF subtype, atrial tissue source, structural heart disease burden, heart failure status, medication exposure, age matching, and inflammatory comorbidities. These factors may confound the interpretation of AF-specific gene-expression changes. Third, although the analyzed genes were categorized as pyroptosis-related based on prior databases and literature, direct evidence of pyroptotic cell death was not established in this study. Functional assays evaluating inflammasome activation, caspase-1 activation, GSDMD cleavage, IL-1β/IL-18 release, and gasdermin-mediated membrane pore formation are necessary. Fourth, ssGSEA based on bulk transcriptomic data provides predicted immune-cell enrichment rather than direct quantification of immune-cell infiltration. Fifth, the recurrence analysis was exploratory because of the limited sample size, incomplete follow-up information, and absence of Cox regression or recurrence-specific ROC analysis. Finally, the ROC curves and drug-gene interaction network were exploratory analyses. The ROC results provide preliminary evidence for the potential discriminatory value of these genes, whereas the predicted drug-gene interactions may serve as a basis for future mechanistic and translational investigations. Further validation in larger cohorts and experimental studies will be necessary to confirm these findings.

## 5. Conclusions

In this study, we integrated transcriptomic analysis, pyroptosis-related gene screening, functional enrichment analysis, external validation, predicted immune-cell enrichment analysis, and clinical sample validation to investigate pyroptosis-related inflammatory signatures in AF. Using the GSE41177 dataset, we identified 472 differentially expressed genes after FDR correction, among which 18 genes overlapped with pyroptosis-related genes. Functional enrichment analyses indicated that these AF-related PRGs were mainly associated with inflammatory and immune-related pathways, including the NOD-like receptor signaling pathway, lipid and atherosclerosis, and infection-related inflammatory pathways. External validation in GSE79768 further supported the diagnostic value of seven candidate genes, namely, *S100A9*, *S100A4*, *MPEG1*, *NAIP*, *CDK9*, *S100A8*, and *S100A12*. ROC analysis showed that these genes had acceptable to excellent discriminatory ability, particularly *CDK9*, *S100A12*, *NAIP*, and *S100A4*. In addition, ssGSEA analysis suggested altered predicted immune-cell enrichment in AF tissues, with higher predicted enrichment of cytotoxic cells, macrophages, mast cells, neutrophils, T cells, Th1 cells, and Treg cells. Finally, qPCR and Western blot analyses confirmed the upregulation of the seven candidate genes in clinical AF samples, and *MPEG1*, *S100A9*, and *S100A4* were further associated with postoperative AF recurrence. Overall, these findings suggest that pyroptosis-related inflammatory gene signatures may be associated with AF and may provide candidate biomarkers and mechanistic hypotheses for future validation.

## Figures and Tables

**Table 1:** Baseline clinical information of control and AF patients.

Variables	Control Group	AF Group	*p* Value
Sex (Male/Female)	5/3	7/9	
Hypertension	4 (50.0%)	5 (31.2%)	
Diabetes mellitus	1 (12.5%)	0 (0.0%)	
Coronary artery disease	1 (12.5%)	1 (6.2%)	
Smoking	0 (0.0%)	3 (18.8%)	
Alcohol consumption	0 (0.0%)	1 (6.2%)	
Age (years)	64.50 ± 6.70	69.62 ± 6.53	0.0968
BMI	24.45 ± 2.17	23.64 ± 3.59	0.4991
LAD (cm)	4.21 ± 0.39	5.74 ± 0.98	<0.001
EF (%)	56.01 ± 4.71	56.46 ± 5.05	0.8324
CRP (mg/L)	7.40 ± 5.35	6.92 ± 6.18	0.8482
ALT (U/L)	28.32 ± 9.27	29.57 ± 12.48	0.7854
TBA (μmol/L)	9.64 ± 4.68	10.93 ± 5.50	0.5575
BUN (mmol/L)	7.26 ± 2.81	8.10 ± 2.51	0.4879

**Table 2:** Baseline clinical information of patients with recurrent and non-recurrent AF.

Variables	Non-Recurrence	Recurrence	*p* Value
Sex (Male/Female)	9/7	8/8	
Hypertension	5 (31.2%)	3 (18.8%)	
Diabetes mellitus	0 (0.0%)	0 (0.0%)	
Coronary artery disease	0 (0.0%)	1 (6.2%)	
Smoking	5 (31.2%)	1 (6.2%)	
Alcohol consumption	3 (18.8%)	0 (0.0%)	
Age (years)	70.38 ± 9.18	67.25 ± 6.52	0.2766
BMI	23.35 ± 3.42	23.61 ± 3.40	0.8272
LAD (mm)	29.70 ± 25.37	26.98 ± 23.00	0.7531
EF (%)	54.91 ± 5.26	54.41 ± 5.54	0.7942
CRP (mg/L)	7.21 ± 5.89	5.89 ± 5.27	0.5098
ALT (U/L)	37.97 ± 20.53	41.97 ± 17.29	0.5550
TBA (μmol/L)	16.43 ± 15.97	12.92 ± 11.35	0.4796
BUN (mmol/L)	8.59 ± 2.23	8.94 ± 3.42	0.7386

## Data Availability

The microarray datasets analyzed during the current study are publicly available in the Gene Expression Omnibus (GEO) under accession numbers GSE41177 and GSE79768. Pyroptosis-related genes (PRGs) were obtained from GeneCards. Gene sets for enrichment analyses were retrieved from MSigDB. Additional interaction and regulatory data were sourced from STRING, TarBase v9.0, ENCODE, DGIdb, and GSVA. Analysis scripts are available from the corresponding author upon reasonable request.

## Supplementary Materials

The following supporting information can be downloaded at: https://www.mdpi.com/article/10.3390/genes17080858/s1. Table S1. Primer sequences used for qPCR. Table S2. Antibody used for WB. Table S3. Baseline clinical information of control and AF patients. Table S4. Baseline clinical information of patients with recurrent and non-recurrent AF.
