# Supplementary material for: Identification of Pyroptosis-Related Gene Signatures Associated with Predicted Immune-Cell Enrichment in Atrial Fibrillation
Source: Genes (Basel). 2026 Jul 24;17(8):858. doi: 10.3390/genes17080858 (PMC13511956; doi:10.3390/genes17080858)
Supplement: Supplementary file 1 [file genes-17-00858-s001.zip › genes-4378335-supplementary.pdf]

Supplementary Materials:

Table S1. Primer sequences used for qPCR.

Gene	Forward primer	Reverse primer
S100A4	GATGAGCAACTGACACAA	CTGGGCTGCTTATCTGGGAAG
S100A8	ATGCCGTCTACAGGGATGAC	ACAGCATCATGAGGTCCTGG
S100A9	TGGAGGACCTGGACACAAATG	CTTTGCCATCAGCATCATACAC
S100A12	CAGCTGGAGAAGGAGCTACT	GCTGCCACGCCCATCTTTAT
MPEG1	AGCTGCTGCTGATGTTCTTC	CAGGATGAGGTCGATGTTGG
NAIP	AGGAGGAGATGCTGCTGAAG	CTGCTTGGTGATGGTGTTGT
CDK9	TTTGCCAGAGCAGATGTTCC	CAGGCTGCTTGGAGATGAAG

Table S2. Antibody used for WB.

Antigen	Manufacturer	Catalog No.
S100A9	Abclonal	A26782PM
S100A4	Abclonal	A19109
MPEG1	Abclonal	A28420
NAIP	Abclonal	A19730
CDK9	Abclonal	A11145
S100A8	Abclonal	A12018
S100A12	Abclonal	A23912

Table S3. Baseline clinical information of control and AF patients

Variables	Control group	AF group	P value
Sex (Male/Female)	5/3	7/9	
Hypertension	4 (50.0%)	5 (31.2%)	
Diabetes mellitus	1 (12.5%)	0 (0.0%)	
Coronary artery disease	1 (12.5%)	1 (6.2%)	
Smoking	0 (0.0%)	3 (18.8%)	
Alcohol consumption	0 (0.0%)	1 (6.2%)	
Age (years)	64.50 ± 6.70	69.62 ± 6.53	0.0968
BMI	24.45 ± 2.17	23.64 ± 3.59	0.4991
LAD (cm)	4.21 ± 0.39	5.74 ± 0.98	<0.001
EF (%)	56.01 ± 4.71	56.46 ± 5.05	0.8324
CRP (mg/L)	7.40 ± 5.35	6.92 ± 6.18	0.8482
ALT (U/L)	28.32 ± 9.27	29.57 ± 12.48	0.7854
TBA (μmol/L)	9.64 ± 4.68	10.93 ± 5.50	0.5575
BUN (mmol/L)	7.26 ± 2.81	8.10 ± 2.51	0.4879

Table S4. Baseline clinical information of patients with recurrent and non-recurrent AF

**Variables**	**Non-recurrence**	**Recurrence**	**P value**
Sex (Male/Female)	9/7	8/8	
Hypertension	5 (31.2%)	3 (18.8%)	
Diabetes mellitus	0 (0.0%)	0 (0.0%)	
Coronary artery disease	0 (0.0%)	1 (6.2%)	
Smoking	5 (31.2%)	1 (6.2%)	
Alcohol consumption	3 (18.8%)	0 (0.0%)	
Age (years)	70.38 ± 9.18	67.25 ± 6.52	0.2766
BMI	23.35 ± 3.42	23.61 ± 3.40	0.8272
LAD(cm)	29.70 ± 25.37	26.98 ± 23.00	0.7531
EF(%)	54.91 ± 5.26	54.41 ± 5.54	0.7942
CRP (mg/L)	7.21 ± 5.89	5.89 ± 5.27	0.5098
ALT(U/L)	37.97 ± 20.53	41.97 ± 17.29	0.5550
TBA(μmol/L)	16.43 ± 15.97	12.92 ± 11.35	0.4796
BUN(mmol/L)	8.59 ± 2.23	8.94 ± 3.42	0.7386
